# Dopamine Is a Siderophore-Like Iron Chelator That Promotes *Salmonella enterica* Serovar Typhimurium Virulence in Mice

**DOI:** 10.1128/mBio.02624-18

**Published:** 2019-02-05

**Authors:** Stefanie Dichtl, Egon Demetz, David Haschka, Piotr Tymoszuk, Verena Petzer, Manfred Nairz, Markus Seifert, Alexander Hoffmann, Natascha Brigo, Reinhard Würzner, Igor Theurl, Joyce E. Karlinsey, Ferric C. Fang, Günter Weiss

**Affiliations:** a Department of Internal Medicine II (Infectious Diseases, Immunology, Rheumatology and Pneumology), Medical University of Innsbruck, Innsbruck, Austria; b Christian Doppler Laboratory for Iron Metabolism and Anemia Research, Medical University of Innsbruck, Innsbruck, Austria; c Division of Microbiology and Hygiene, Medical University of Innsbruck, Innsbruck, Austria; d Department of Laboratory Medicine, University of Washington School of Medicine, Seattle, Washington, USA; e Department of Microbiology, University of Washington School of Medicine, Seattle, Washington, USA; UTMB; UT Southwestern Medical Center Dallas

**Keywords:** *Salmonella* Typhimurium, catecholamine, dopamine, iron, *qseC*, sepsis

## Abstract

We have recently shown that the catecholamine dopamine regulates cellular iron homeostasis in macrophages. As iron is an essential nutrient for microbes, and intracellular iron availability affects the growth of intracellular bacteria, we studied whether dopamine administration impacts the course of *Salmonella* infections. Dopamine was found to promote the growth of *Salmonella* both in culture and within bone marrow-derived macrophages, which was dependent on increased bacterial iron acquisition. Dopamine administration to mice infected with Salmonella enterica serovar Typhimurium resulted in significantly increased bacterial burdens in liver and spleen, as well as reduced survival. The promotion of bacterial growth by dopamine was independent of the siderophore-binding host peptide lipocalin-2. Rather, dopamine enhancement of iron uptake requires both the histidine sensor kinase QseC and bacterial iron transporters, in particular SitABCD, and may also involve the increased expression of bacterial iron uptake genes. Deletion or pharmacological blockade of QseC reduced but did not abolish the growth-promoting effects of dopamine. Dopamine also modulated systemic iron homeostasis by increasing hepcidin expression and depleting macrophages of the iron exporter ferroportin, which enhanced intracellular bacterial growth. *Salmonella* lacking all central iron uptake pathways failed to benefit from dopamine treatment. These observations are potentially relevant to critically ill patients, in whom the pharmacological administration of catecholamines to improve circulatory performance may exacerbate the course of infection with siderophilic bacteria.

## Abstract (precis)

Here we show that dopamine increases bacterial iron incorporation and promotes *Salmonella* Typhimurium growth both *in vitro* and *in vivo*. These observations suggest the potential hazards of pharmacological catecholamine administration in patients with bacterial sepsis but also suggest that the inhibition of bacterial iron acquisition might provide a useful approach to antimicrobial therapy.

## INTRODUCTION

Iron is essential for eukaryotes but also for nearly all microorganisms as a central component of many proteins involved in metabolic functions and cellular proliferation (1, 2). Thus, during infection, a coordinated host response limits the availability of iron to microbes. Mechanisms of iron withholding are dependent on the localization and nature of the specific pathogens and immune cells involved (3–5). Iron limitation restricts the replication of invading pathogens in a process known as “nutritional immunity” (6). During infection with extracellular bacteria, cytokines and hepcidin, the master regulator of iron homeostasis, promote macrophage iron accumulation and retention, resulting in hypoferremia, anemia of inflammation, and a reduction in the availability of tissue iron (4, 7). In contrast, infection with intracellular bacteria, such as *Salmonella* and Mycobacteria, leads to an increased iron egress from macrophages in order to limit intracellular iron (8). As a result of interactions between iron homeostasis and immune function, changes in iron availability also influence the expression of antimicrobial effector pathways and the differentiation and proliferation of immune cells involved in host control of infection (5, 9). The spatiotemporal redistribution of divalent metals is an important determinant of host resistance (10, 11). Thus, the mechanisms of systemic and cellular iron homeostasis have a major influence on the course and outcome of infection. Salmonella enterica is a highly iron-dependent intracellular Gram-negative bacterial pathogen with more than 2,500 different serovars, which can cause local intestinal disease or severe systemic infection and septicemia (12). Salmonella enterica is responsible for an estimated one million deaths annually (13). Due to increased multidrug resistance, the WHO has included *Salmonella* in the list of the most serious infectious disease threats to human health. *Salmonella* has both siderophore-dependent and -independent strategies to acquire iron from the host (14). *Salmonella* synthesizes catecholate-type siderophores such as enterochelin and salmochelin, a C-glucosylated enterobactin, to capture and internalize ferric iron via siderophore receptors (15–17). In addition to bacterium-derived siderophores like enterobactin, other catechols can serve as pseudosiderophores that are able to promote bacterial growth under iron-restricted conditions (18). Catecholamines are stress hormones that can interact with transferrin-bound Fe(III) and promote its reduction to Fe(II), for which Tf has little affinity (19). We have recently shown that the catecholamine dopamine (DA) impacts the iron homeostasis of macrophages, promoting cellular iron accumulation in macrophages by a poorly understood mechanism and stimulating intracellular antistress responses (20). This is of interest because previous studies have shown that catecholamines can promote the growth of various pathogenic bacteria, including Staphylococcus aureus, Listeria monocytogenes, Escherichia coli, Yersinia enterocolitica, and Salmonella enterica (21–23). Sandrini et al. found that clinically relevant concentrations of DA can compromise the iron-binding integrity of Tf and thereby enable proliferation of invading bacteria by making serum less bacteriostatic (19). In clinical practice, catecholamines are a cornerstone for the treatment of critically ill patients, including those with septic shock, where they are used to stabilize the circulatory system. However, catecholamines can also bind to two histidine sensor kinases QseC and QseE, resulting in effects on bacterial proliferation and virulence (24). The transcription of *qseE* is activated by QseC; therefore, QseC acts upstream of QseE (25). QseC regulates the transcription of *Salmonella* pathogenicity island 1 (SPI-1) genes, the SPI-2 effector locus *sifA*, and flagellar genes, both *in vivo* and *in vitro* (26, 27). In various pathogens, a small molecule inhibitor of QseC called LED209 was described (28, 29). The prodrug LED209 does not interfere with pathogen growth and may therefore exert less evolutionary pressure favoring the development of drug resistance (30). Here, we provide novel evidence that the catecholamine DA stimulates the proliferation and intramacrophage survival of *Salmonella enterica* serovar Typhimurium and worsens the course of *Salmonella* infections by serving as an iron source for these bacteria.

## RESULTS

### Dopamine promotes *S*. Typhimurium growth *in vitro*.

**FIG 1:**
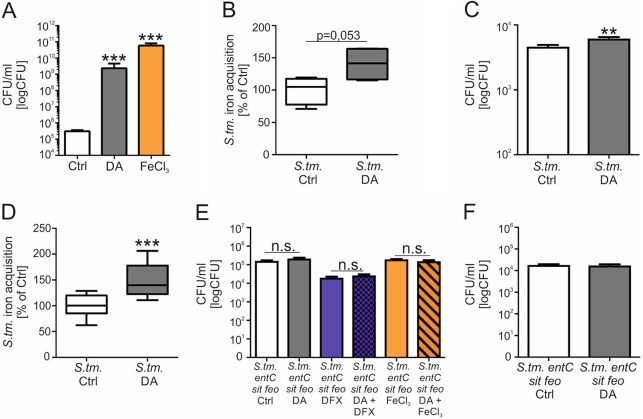
Dopamine promotes *S.* Typhimurium growth *in vitro. S.* Typhimurium (*S*. tm.) was cultured in the presence of 5 µM DA, 1 µM FeCl_3_, or solvent (Ctrl) for 12 h in DMEM, and bacterial load was determined by plating (*n* = 3 independent experiments) (A). Values were log transformed, and results were compared by *t* test. Superscripts indicate statistical significance compared to the control group. *S.* Typhimurium bacteria were cultured with ^59^Fe and, where indicated, with 5 µM DA in RPMI medium for 3 h (B). Bacterial iron acquisition was determined by quantification of ^59^Fe in a gamma counter. Data are shown as means ± SEMs from three independent experiments and expressed as percentage of *S*. Typhimurium iron acquisition compared with the control (100%). Wt BMDMs were infected with *S.* Typhimurium (C) (*n* = 4 independent experiments) or *S.* Typhimurium lacking *entC*, *sit*, and *feo* genes (*S*. tm. *entC sit feo*) (F) (*n* = 3 independent experiments) and stimulated with DA or solvent for 12 h. Bacterial numbers were calculated by plating. Values were log transformed, and results were compared by *t* test. Wt BMDMs were infected with *S.* Typhimurium, stimulated with ^59^Fe, and, where indicated, treated with DA (5 µM) for 12 h (D). Bacterial iron acquisition was determined by quantification of ^59^Fe in a gamma counter. Data are shown as means ± SEMs from three independent experiments and expressed as percentage of *S*. Typhimurium iron acquisition compared with control (100%). Values are depicted as lower quartile, median, and upper quartile (boxes) with minimum and maximum ranges. *S.* Typhimurium lacking *entC*, *sit*, and *feo* genes (*S*. tm. *entC sit feo*) was grown in the presence of 5 µM DA, 50 µM DFX, 1 µM FeCl_3_, or solvent (Ctrl) for 12 h in DMEM, and bacterial load was determined by plating (*n* = 3 independent experiments) (E). Values were log transformed, and results were compared by *t* test. **, *P* < 0.01; ***, *P* < 0.001.

To study the effects of DA on *S.* Typhimurium growth, bacterial growth was measured in the presence of DA or FeCl_3_ as a positive control (Fig. 1A; see also Fig. S1A in the supplemental material). After 12 h of incubation, significantly higher *Salmonella* numbers were found after addition of DA compared to bacteria cultured without DA. Notably, the bacterial growth-promoting effect of DA was comparable to that observed after supplementation with FeCl_3_. As we have previously observed that DA increases iron accumulation in macrophages (20), we questioned whether increased iron delivery to bacteria might account for higher bacterial numbers. Therefore, we measured ^59^Fe acquisition by *S.* Typhimurium and found that after 3 h of DA exposure, cultured bacteria exhibited an approximately 40% increase in iron acquisition in comparison to bacteria grown in the absence of DA (Fig. 1B). To determine if this was relevant to intracellular *Salmonella*, we infected BMDMs from C57BL/6 mice with *S.* Typhimurium at an MOI of 10:1 for 12 h. During infection, DA was added in the presence of tranylcypromine, a monoamine oxidase inhibitor that prevents DA degradation (Fig. S1B) (20). The addition of DA resulted in significantly increased intramacrophage numbers of *S.* Typhimurium in comparison to infected macrophages without added DA serving as a control (Fig. 1C). The addition of tranylcypromine without DA had no effect on bacterial numbers compared to controls (results not shown). Furthermore, iron acquisition by *Salmonella* was found to be significantly enhanced in infected BMDMs treated with DA, in contrast to macrophages without the addition of DA (Fig. 1D). To determine whether the effect of DA on *S.* Typhimurium growth is due to increased iron delivery, the growth of *S.* Typhimurium lacking *entC*, *sit*, and *feo*, three central iron uptake systems of *S.* Typhimurium, was monitored in the presence of DA. EntC is required for synthesis of the siderophores enterobactin and salmochelin, the ATP-binding cassette transporter SitABCD mediates the uptake of ferrous iron and manganese (31–34), and the FeoAB system transports ferrous iron (35). The addition of DA had no effect on the growth of *S.* Typhimurium lacking *entC*, *sit*, and *feo* in either iron-depleted (DFX-treated) or iron-supplemented medium (Fig. 1E). Using this mutant strain of *S.* Typhimurium, a stimulatory effect of DA on intracellular *S.* Typhimurium growth was no longer observed (Fig. 1F). These data indicate that DA stimulates intracellular *S.* Typhimurium growth by increasing bacterial iron acquisition.

10.1128/mBio.02624-18.2 FIG S1 *S*. Typhimurium (*S*. tm.) was grown in the presence of 500 nM, 5 µM, or 50 µM DA or solvent (Ctrl) for 12 h in DMEM, and bacterial load was determined by plating (*n* = 2 independent experiments) (A). Values were log transformed, and the results were compared by *t* test. Superscripts indicate statistical significance compared to the control group. *S*. tm. was grown in the presence of 5 µM DA (*S*. tm. DA), 5 µM DA plus 100 nM tranylcypromine (*S*. tm. DA + T), or solvent (Ctrl) for 12 h in DMEM, and bacterial load was determined by plating (*n* = 2 independent experiments) (B). Healthy Wt mice were injected with DA or solvent (Ctrl) every 12 h, and spleens were analyzed for *iNOS* (C) and *Lcn2* (D) expression. Data were normalized for mRNA levels of *HPRT*. Wt mice were injected with DA or solvent (Ctrl) every 12 h and, where indicated, i.p. infected with *S*. Typhimurium (*S*. tm.). Cells were first gated using FSC/SSC characteristics, and doublets were sequentially excluded by comparing FSC width and area signals (E). Red pulp macrophages (RPMs) were identified as CD45^+^, Lin^−^ (Lin = CD3, CD19, CD49b) Gr1^−^, CD11b^low/dim^, F4/80^high^. *S*. Typhimurium (*S*. tm.) or an isogenic *qseC* mutant strain (*S*. tm. *qseC*) was grown in the presence of 5 µM DA or solvent (Ctrl) for 12 h in DMEM, and the expression of bacterial iron metabolic genes was measured by qRT-PCR. Expression of *fur* (F) and *fepC* (G) was determined relative to the housekeeping gene *gyrB*. Superscripts indicate statistical significance compared to the control group. Download FIG S1, PDF file, 4.2 MB. Copyright © 2019 Dichtl et al. 2019 Dichtl et al. This content is distributed under the terms of the Creative Commons Attribution 4.0 International license.

### Dopamine worsens the outcome of *S.* Typhimurium infection *in vivo*.

**FIG 2:**
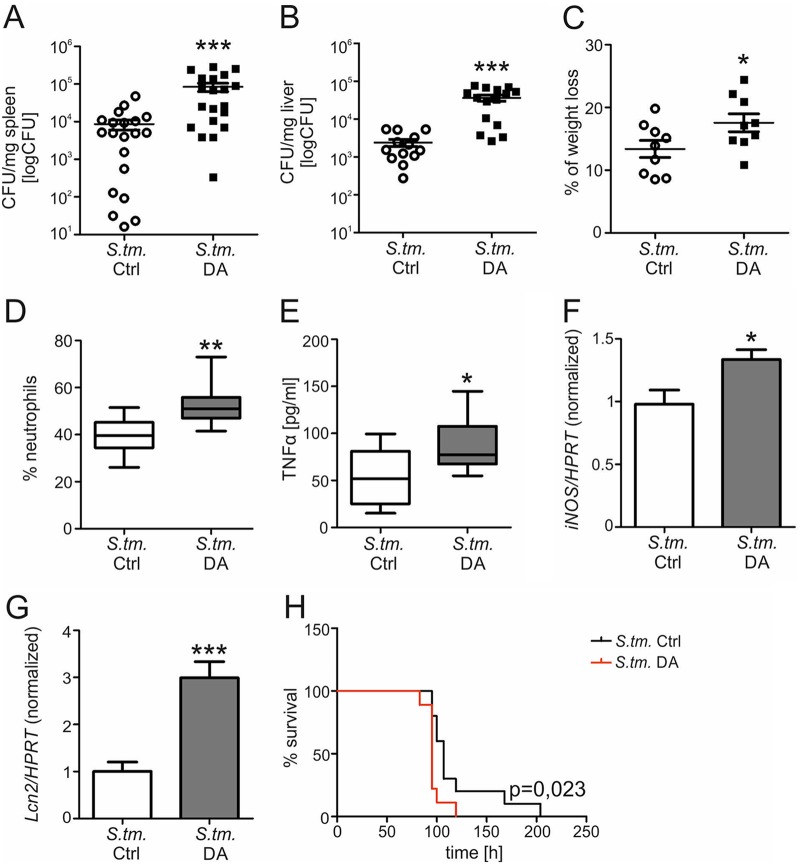
Dopamine worsens the outcome of *S.* Typhimurium infection *in vivo.* Wt mice were infected with *S.* Typhimurium (*S*. tm.) and injected with DA or solvent (Ctrl) every 12 h. After 72 h, the bacterial load in spleens (A) (*n* = 20 individual mice per group) and livers (B) (*n* = 13 versus 15) was determined by plating serial dilutions of tissue lysates. Each dot shows an individual mouse; bars indicate means. Values were log transformed, and results were compared by *t* test. Wt mice were infected with *S*. Typhimurium and treated with DA or solvent every 12 h. After 72 h, the weight loss in comparison to the weight before infection was determined (*n* = 9 individual mice per group) (C). The percentage of neutrophils in blood was detected (*n* = 9 individual mice per group) (D). Values are depicted as lower quartile, median, and upper quartile (boxes) with minimum and maximum ranges. The concentration of TNF-α in serum was measured by ELISA (*n* = 9 individual mice per group) (E). Spleens were analyzed for *iNOS* (F) (*n* = 8 versus 7) and *Lcn2* (G) (*n* = 10 versus 9) expression. Data were normalized for mRNA levels of HPRT, and relative changes compared to solvent-treated mice are shown. Values are depicted as lower quartile, median, and upper quartile (boxes) with minimum and maximum ranges. For survival experiments, C57BL/6 mice were infected and treated until termination occurred (H). The representative Kaplan-Meier curve displays mouse survival over time. Statistically significant differences were determined by log-rank test (*n* = 10 versus 9). Statistically significant differences were determined by *t* test. *, *P* < 0.05; **, *P* < 0.01; ***, *P* < 0.001.

Based on these results, we investigated the effects of DA on the course of *S.* Typhimurium infection *in vivo*. Wt mice were infected with *S.* Typhimurium i.p. to induce a systemic infection and then injected i.p. with DA or solvent every 12 h. After 72 h, the mice were sacrificed and bacterial numbers in spleens (Fig. 2A) and livers (Fig. 2B) were evaluated. DA-treated mice showed a significantly increased bacterial load in spleens and livers. Accordingly, DA-treated mice exhibited increased weight loss during *S.* Typhimurium infection (Fig. 2C) and significantly enhanced numbers of neutrophils (Fig. 2D), both indicative of more severe infection. We then determined whether DA treatment might dampen immune response pathways, which are of importance in the control of *Salmonella* infection. However, circulating levels of the cytokine TNF-α were actually significantly increased in infected mice receiving DA (Fig. 2E), and the expression of mRNA for inducible nitric oxide synthase (*iNOS*) was significantly higher in spleens of DA-treated mice than in solvent-treated mice (Fig. 2F). As macrophages produce the bacterial siderophore-binding protein Lcn2 to control infection, we also measured *Lcn2* mRNA expression in spleens (Fig. 2G). *S.* Typhimurium-infected mice treated with DA had significantly higher levels of *Lcn2* expression than solvent-treated mice. No difference in the expression of *iNOS* or *Lcn2* was detected in uninfected mice treated with DA (Fig. S1C and D). Collectively, these observations indicate that DA does not impair essential innate antimicrobial immune responses, and the increased immune responses observed in DA-treated mice are likely to result from increased immune stimulation resulting from a higher bacterial burden.

To evaluate whether the growth-promoting effect of DA on intramacrophage *Salmonella* translates into different outcomes, we determined the survival of infected mice treated with DA or solvent every 12 h until termination (Fig. 2H). Mice receiving DA exhibited significantly reduced survival and succumbed to infection approximately 12 h earlier than solvent-treated mice.

### Dopamine influences iron homeostasis during *S.* Typhimurium infection *in vivo*.

**FIG 3:**
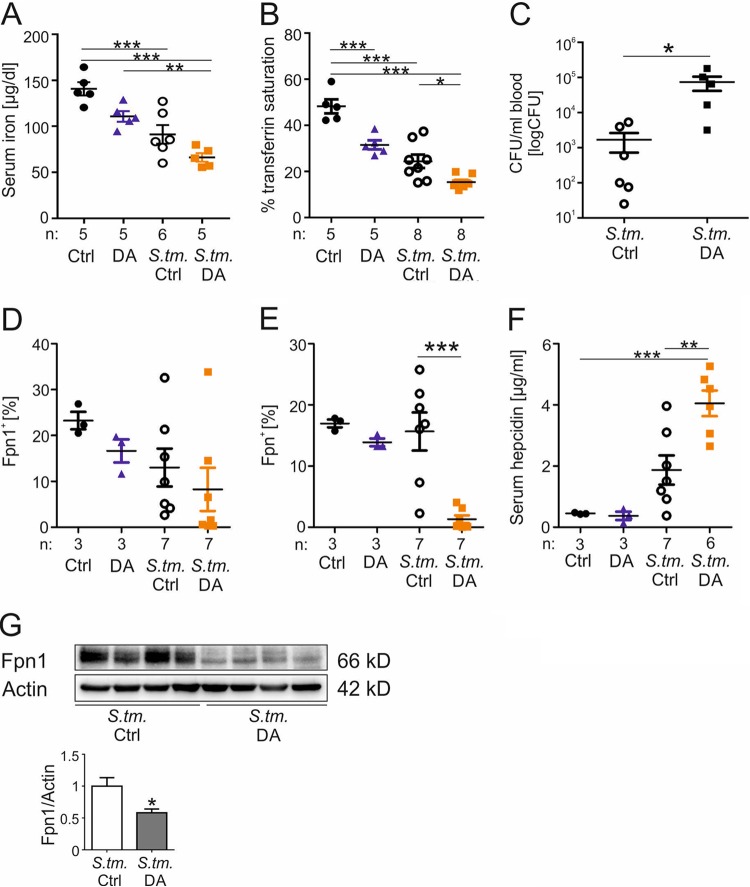
Dopamine influences iron homeostasis during *S.* Typhimurium infection *in vivo.* Wt mice were injected with DA or solvent (Ctrl) every 12 h and, where indicated, i.p. infected with *S.* Typhimurium (*S*. tm.). After 72 h, serum iron concentration was measured (A). Further transferrin saturation was detected by measuring serum iron and Tf concentrations (B). Each dot shows an individual mouse; bars indicate means; “n” indicates the number of mice. After 72 h of infection, bacterial counts in blood were determined by plating serial dilutions (*n* = 6 versus 5) (C). Values were log transformed, and results were compared by *t* test. Percentages of splenic (D) and liver (E) Fpn1^+^ CD45^+^ cells were detected by flow cytometry analysis after 72 h. After 72 h, serum hepcidin concentrations were measured using a commercially available kit (F). Western blot analysis of splenic whole-cell lysates was determined using specific antibodies to Fpn1 and the loading control β-actin (G). Western blot bands were quantified densitometrically. Each sample shows an individual mouse spleen. Statistically significant differences determined by ANOVA using Bonferroni’s correction or *t* test are indicated. *, *P* < 0.05; **, *P* < 0.01; ***, *P* < 0.001.

Having established that DA shortens the survival of septic mice and increases intramacrophage bacterial numbers by promoting iron delivery to *Salmonella*, we examined whether DA influences systemic iron homeostasis in infected mice. Mice were infected with *S.* Typhimurium or left uninfected in the presence or absence of DA for 72 h, as described above, and then sacrificed. Serum iron concentrations were significantly decreased in infected mice receiving DA (Fig. 3A). Transferrin saturation was determined as an indicator of the amount of metabolically available iron in the circulation, as DA has been shown to promote the reduction of ferric to ferrous iron, which is only weakly bound by transferrin (Fig. 3B) (19). DA treatment of uninfected mice caused a significant decrease in transferrin saturation compared to solvent-treated mice. Upon infection with *S.* Typhimurium, transferrin saturation declined significantly, and DA treatment led to a further decrease in transferrin saturation.

While one might predict a bacteriostatic effect of lower transferrin saturation, numbers of circulating *Salmonella* were actually significantly higher in DA-treated mice (Fig. 3C). To investigate the effects of DA treatment on cellular iron homeostasis in uninfected and *S.* Typhimurium-infected mice, we measured the expression of the iron exporter ferroportin (Fpn1) in CD45^+^ cells of spleen and liver. Fpn1 is the only known cellular iron exporter and a target of hepcidin, which promotes Fpn1 internalization and the inhibition of cellular iron egress (36, 37). Moreover, Fpn1 plays a key role in the metabolic response to infections with intracellular bacteria. Fpn1 overexpression has been shown to improve control of the replication of intracellular bacteria, including *Salmonella* (38–40). Analysis of splenic Fpn1^+^ CD45^+^ cells showed a slightly reduced number of Fpn1-expressing cells in DA-treated uninfected mice (Fig. 3D). After infection, the percentage of Fpn1^+^ splenocytes further declined, and DA treatment led to a further decrease in splenic Fpn1^+^ CD45^+^ cells. The latter effect was even more pronounced in CD45^+^ cells of the liver (Fig. 3E; gating strategy shown in Fig. S1E). To determine if decreased Fpn1 expression was linked to upregulation of hepcidin expression, serum hepcidin concentrations were measured. Although there was no difference between DA- and solvent-treated uninfected mice, hepcidin levels increased in *S.* Typhimurium-infected solvent-treated mice (Fig. 3F), and DA administration resulted in a further increase in serum hepcidin levels. To further verify the observed effects of DA on iron homeostasis in infected mice, splenic Fpn1 protein expression was quantified by Western blotting (Fig. 3G). In accordance with the flow cytometry data, Fpn1 expression in DA-treated mice was significantly reduced in comparison to solvent-treated infected mice.

### The bacterial histidine sensor kinase QseC is involved in dopamine-mediated iron uptake by *S.* Typhimurium.

**FIG 4:**
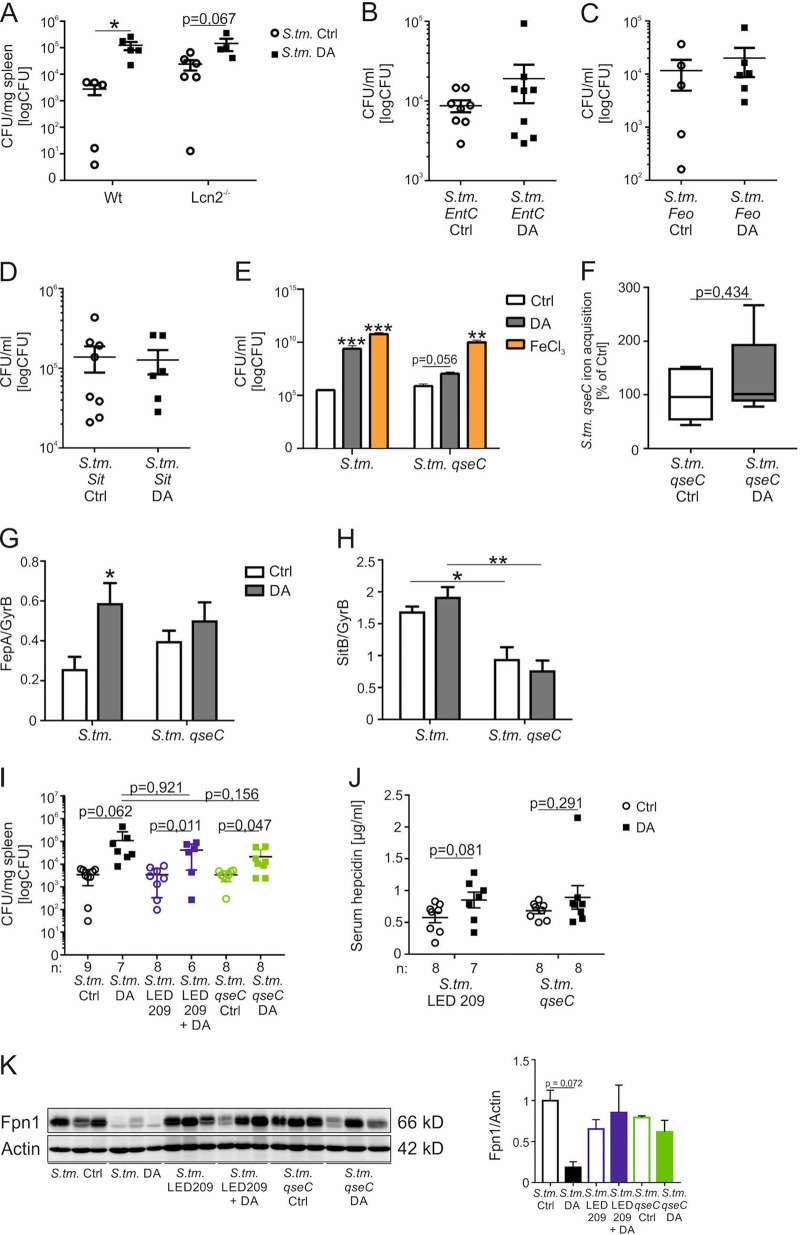
The bacterial histidine sensor kinase QseC is involved in dopamine-mediated iron acquisition by *S.* Typhimurium. Wt mice and Lcn2^−/−^ mice were infected with *S.* Typhimurium (*S*. tm.) and injected with DA or solvent (Ctrl) every 12 h. After 72 h, the bacterial load in spleens was determined by plating serial dilutions of tissue lysates (A). Each dot shows an individual mouse; bars indicate means. Values were log transformed, and statistical significance was determined by the Mann-Whitney test. Wt mice were infected with an *S.* Typhimurium *entC* mutant (*S*. tm. *entC*) (B), *S.* Typhimurium *feo* mutant (*S*. tm. *feo*) (C), or *S.* Typhimurium *sit* mutant (*S*. tm. *sit*) (D) and treated with DA or solvent every 12 h. After 72 h, the bacterial load in spleens was measured by plating serial dilutions of tissue lysates. *S.* Typhimurium (*S*. tm.) and the isogenic *qseC* mutant strain (*S*. tm. *qseC*) were grown in the presence of 5 µM DA, 1 µM FeCl_3_, or solvent (Ctrl) for 12 h in DMEM, and bacterial load was determined by plating (*n* = 3 independent experiments) (E). Values were log transformed, and results were compared by *t* test. Superscripts indicate statistical significance compared to the control group. Wt BMDMs were infected with an *S.* Typhimurium *qseC* mutant, stimulated with ^59^Fe, and, where indicated, treated with DA for 12 h (F). Bacterial iron acquisition was determined by quantification of ^59^Fe in a gamma counter. Data are shown as means ± SEMs from three independent experiments and expressed as percentage of *S*. Typhimurium *qseC* iron acquisition compared with control (100%). Values are depicted as lower quartile, median, and upper quartile (boxes) with minimum and maximum ranges. *S.* Typhimurium (*S*. tm.) or an isogenic *qseC* mutant strain (*S*. tm. *qseC*) was grown in the presence of 5 µM DA or solvent (Ctrl) for 12 h in DMEM, and the expression of bacterial iron metabolic genes was measured by qRT-PCR. Expression of *fepA* (G) and *sitB* (H) was determined relative to the housekeeping gene *gyrB*. Wt mice were injected with DA or solvent every 12 h and i.p. infected with Wt *S.* Typhimurium (*S*. tm.) or an *S.* Typhimurium *qseC* mutant (*S*. tm. *qseC*). Where indicated, the mice were treated with LED209. After 72 h, the bacterial load in spleens was determined by plating serial dilutions of tissue lysates (I). Each dot shows an individual mouse; bars indicate means; “n” indicates the number of mice. Serum hepcidin concentrations were measured using a commercially available kit (J). Western blot analysis of splenic whole-cell lysates was determined using specific antibodies to Fpn1 and the loading control β-actin (K). Western blot bands were quantified densitometrically. Statistically significant differences determined by ANOVA using Bonferroni’s correction or *t* test are indicated.

In view of the higher expression of Lcn2 in DA-treated mice, we wondered whether the siderophore-binding host protein Lcn2 plays an important role in the increased bacterial burden of DA-treated mice. We therefore infected Wt or Lcn2^−/−^ mice with *S.* Typhimurium and treated them with DA every 12 h. DA treatment caused an increased bacterial load in the spleens in both groups, but no significant difference in bacterial numbers was observed between Wt and Lcn2^−/−^ mice (Fig. 4A). Therefore, Lcn2 does not appear to be responsible for the effects of DA on intracellular bacterial growth.

To further confirm the role of iron uptake systems in DA-mediated enhancement of bacterial growth, we infected mice with isogenic *Salmonella entC* (Fig. 4B), *feo* (Fig. 4C), or *sitABCD* (Fig. 4D) single mutant strains and treated them with DA every 12 h. For each of these mutant strains, DA treatment no longer caused a significant increase in bacterial numbers compared to solvent-treated mice. Nevertheless, a trend toward higher bacterial burdens was still observed in DA-treated mice infected with *entC* or *feo* mutant *Salmonella* but not in DA-treated mice infected with *sitABCD* mutant *Salmonella*. This suggests that DA-mediated bacterial iron uptake may principally involve the SitABCD transport system.

To determine whether the bacterial histidine sensor kinase QseC is required for DA stimulation of bacterial growth, we measured the CFU of Wt and *qseC* mutant *Salmonella* in the presence of DA and FeCl_3_ (Fig. 4E). In contrast to the Wt *Salmonella* strain, DA failed to stimulate growth of the *qseC* mutant, indicating that QseC is required for the stimulation of bacterial growth by DA. To confirm the role of QseC in DA-dependent iron uptake, we measured iron acquisition by an *S.* Typhimurium *qseC* mutant in the presence or absence of DA (Fig. 4F). Wt BMDMs infected with the *S.* Typhimurium *qseC* mutant showed comparable bacterial iron acquisition in the presence and absence of DA, suggesting that the histidine sensor kinase QseC is important for DA-dependent iron acquisition by *S.* Typhimurium. To determine whether DA-QseC interactions influence the expression of iron uptake genes, we measured the mRNA of iron-regulated genes in cultured *S.* Typhimurium. DA of Wt *S.* Typhimurium increased the expression of *fepA*, encoding a siderophore receptor (Fig. 4G) (41), which required the presence of QseC. Furthermore, decreased expression of *sitB* was observed in *qseC* mutant *S.* Typhimurium in comparison to Wt (Fig. 4H). Interestingly, not all iron-regulated genes were modulated by QseC; for example, *fepC* (Fig. S1G) and *fur* (Fig. S1F) expression are QseC independent. These observations suggest that QseC has a complex and variable influence on iron-regulated genes, as has been reported in E. coli (42).

To explore the role of QseC in DA stimulation of bacterial growth in mice, Wt mice were infected with *S.* Typhimurium or an isogenic *S.* Typhimurium mutant lacking the histidine sensor kinase QseC. Additional Wt mice were infected with Wt *S.* Typhimurium and orally treated with two doses of LED209, a pharmacological inhibitor of QseC. Infection of mice with Wt *S.* Typhimurium in the presence or absence of LED209 did not result in different bacterial numbers, which was also observed in mice infected with an *S.* Typhimurium *qseC* mutant strain. However, although DA administration resulted in increased bacterial numbers in all three groups, bacterial loads were lower by trend in mice infected with the *qseC* mutant compared with Wt *S.* Typhimurium (Fig. 4I).

To investigate this possibility further, serum hepcidin was measured in mice infected with *qseC* mutant or Wt *S.* Typhimurium in the presence or absence of LED209. In contrast to the experiment shown in Fig. 3F, we could not detect a DA-dependent increase in serum hepcidin concentrations in mice infected with the *S.* Typhimurium *qseC* mutant, nor in Wt *S.* Typhimurium-infected mice receiving the pharmacological QseC inhibitor LED209 (Fig. 4J). These results were verified by measuring splenic Fpn1 protein levels. As shown earlier, DA administration resulted in decreased Fpn1 expression in Wt *S.* Typhimurium-infected mice. However, this was not observed in mice infected with *qseC* mutant *S.* Typhimurium or in mice receiving LED209 (Fig. 4K). These observations indicated that DA-QseC interactions are important for the modulation of host cellular iron homeostasis by *Salmonella*. This may contribute to the influence of DA on bacterial burden, as DA effects on the ferroportin-hepcidin axis can increase iron availability for intramacrophage bacteria.

## DISCUSSION

Dopamine (DA) is a neurotransmitter in the catechol family, which is often used as first-line vasoactive drug in patients with septic shock (43). However, adverse events are more frequently associated with DA treatment in comparison to the closely related molecule norepinephrine. As DA and norepinephrine differ with regard to their effects on macrophage iron homeostasis (20), we hypothesized that DA might influence the control of infection with siderophilic bacteria such as *Salmonella* (22).

Specifically, we found that DA increases iron acquisition by *S.* Typhimurium, which leads to enhanced proliferation, higher bacterial numbers in tissues, and significantly reduced survival of infected mice. The effects of DA on bacterial proliferation are abrogated by mutation of the *Salmonella entC*, *sit*, and *feo* genes, which encode important iron uptake systems, indicating that iron is involved in DA stimulation of bacterial proliferation. DA does not directly influence essential antimicrobial innate immune responses, which rather are enhanced as a result of higher bacterial numbers and more sustained immune stimulation.

DA appears to promote bacterial growth by at least two mechanisms, both of which increase bacterial iron availability. First, DA is able to bind iron directly as a siderophore-like molecule to facilitate *Salmonella* iron uptake. Even in the absence of macrophages, DA has a growth-promoting effect on *S.* Typhimurium which is comparable to that of FeCl_3_. Furthermore, in the presence of QseC, DA can increase the expression of bacterial iron uptake genes like *fepA*. These findings suggest that DA-QseC signaling may modulate the expression of specific *Salmonella* iron transport genes, as previously reported in E. coli (42). The stimulation of iron acquisition by DA may reflect a combination of pseudosiderophore-mediated iron delivery and altered expression of iron transport genes, in particular the SitABCD system.

Although we found that the stimulatory actions of DA are not related to expression of the siderophore-binding protein Lcn2, DA does influence systemic host iron homeostasis. We found that DA treatment causes a significant reduction in the number of Fpn1^+^ CD45^+^ cells in the liver, which is paralleled by reduced Fpn1 expression. Reduced Fpn1 expression results in impaired immune control of intracellular pathogens as a result of intramacrophage iron retention and increased iron accessibility (26, 39, 44, 45). This appears to be the result of increased expression of the master iron regulator hepcidin, which promotes Fpn1 internalization and degradation (1, 36). Interestingly, the deletion of QseC blunted this hepcidin-inducing effect, suggesting that *Salmonella* may contribute to hepcidin induction, possibly via stimulation of estrogen-related receptor γ signaling in the liver (46). Moreover, DA stimulation results in macrophage iron accumulation (20) and increased acquisition of non-transferrin-bound iron by an as-yet-uncharacterized mechanism, which also promotes intracellular bacterial growth.

The conventional treatment of septic shock includes vasoactive drugs such as DA. *Salmonella* displays one of the most serious infectious disease threats for human health. Our findings suggest that the use of DA as a vasoactive drug in patients with septic shock due to infection with intracellular pathogens such as *Salmonella* might have adventitious effects on iron homeostasis and bacterial growth that are detrimental to the host. On the other hand, our observations suggest that interventions to limit microbial iron access represent a promising strategy for the treatment of infection.

## MATERIALS AND METHODS

### Bacterial strains and mutant constructions.

Salmonella enterica serovar Typhimurium Wt strain 14028 was obtained from the American Type Culture collection (ATCC, Manassas, VA, USA). Isogenic mutant strains lacking the primary *Salmonella* iron transport systems contained *entC*::*aph* (47)*, Δsit*::*bla* (31), or *Δfeo*::Tn*10* (31) mutations, which were combined by phage P22-mediated transduction to create strain MLC774, lacking all three transport systems. *S.* Typhimurium *qseC* mutant JK1435 was constructed as described by Datsenko and Wanner (48) with primers JKP800 (5′-GCCTCAAATCCACCTTCCGCGGCGTTGCCAAACGACACCGGTGTAGGCTGGAGCTGCTTC) and JKP801 (5′-GTCTCAGCCTGCGCGTCAGGCTGACGCTTATTTTCCTGATCATATGAATATCCTCCTTAG).

### Cell isolation and culture.

Bone marrow-derived macrophages (BMDMs) were harvested from wild-type (Wt) C57BL/6 mice. The mice were housed in neighboring cages and kept under pathogen-free conditions at the central animal facility of the Medical University of Innsbruck. Tibias and femurs were collected and flushed with cold PBS containing 1% penicillin-streptomycin as described previously (20). BMDMs were cultured in DMEM for 7 days in the presence of 50 ng/ml recombinant murine M-CSF (Peprotech, Vienna, Austria).

### *Salmonella* infection *in vitro*.

BMDMs were washed several times with PBS and incubated in DMEM without antibiotics. Salmonella enterica serovar Typhimurium Wt strain ATCC 14028 (*S.* Typhimurium) or an isogenic mutant strain deficient for central pathways of iron uptake (*entC*::*aph*, *Δsit*::*bla*, and *Δfeo*::Tn*10*) (*S*. tm. *entC sit feo*) was used for experiments and grown under sterile conditions in LB broth (Sigma-Aldrich, Vienna, Austria) to late logarithmic phase (47). BMDMs were infected with *S.* Typhimurium at a multiplicity of infection (MOI) of 10 for 1 h. After incubation with *S.* Typhimurium, BMDMs were washed with PBS and incubated in complete DMEM containing gentamicin (Life Technologies, NY, USA). Where indicated, medium was supplemented with 5 µM DA (Sigma-Aldrich, Vienna, Austria) and 100 nM tranylcypromine (Sigma-Aldrich, Vienna, Austria) for up to 12 h. Control samples were treated with solvent. To determine intracellular bacterial loads, infected BMDMs were harvested in 0.5% sodium deoxycholic acid (Sigma-Aldrich, Vienna, Austria) as described previously (49).

### Bacterial growth assay.

*S.* Typhimurium was grown under sterile conditions in LB broth to late logarithmic phase. Bacteria were counted, and 10^4^ *S.* Typhimurium bacteria were incubated in DMEM. Where indicated, 5 µM DA, 50 µM desferrioxamine (DFX), or 1 µM FeCl_3_ (50) was added to the bacterial suspension. Bacterial inocula were incubated in a shaking platform at 37°C for 12 h, and bacterial colony numbers were determined by plating.

### Bacterial iron acquisition.

Iron uptake by intracellular *S.* Typhimurium was measured according to a modified protocol (38, 51). *S.* Typhimurium was cultured with 10 μM ^59^Fe-citrate for 3 h in RPMI medium. Wt BMDMs were infected with *S.* Typhimurium or an isogenic *S.* Typhimurium *qseC* mutant at an MOI of 20. A 10 μM concentration of ^59^Fe (Perkin-Elmer) dissolved in trisodium -citrate (Sigma-Aldrich, Vienna, Austria) was added to infected and uninfected control macrophages. After incubation for 12 h, cells were washed with 0.9% NaCl containing FeCl_3_ (50 μM), and washing buffer was added to lyse the cells. An aliquot of the suspension was kept for assessment of total iron and bacterial load (51). One thousand units of DNase I/ml (Roche Diagnostics, Mannheim, Germany) was added and centrifuged before the bacterial supernatant was loaded onto 0.22-μm PVDF filters (Merck, Darmstadt, Germany). The filters were centrifuged, washed, and measured by a gamma counter.

### *Salmonella* infection *in vivo*.

All animal experiments were performed according to institutional and governmental guidelines in the animal facility of the Medical University of Innsbruck. Design of the animal experiments was approved by the Austrian Federal Ministry of Science and Research (BMWF-66.011/0092-II/3b/2013). Male C57BL/6N Wt mice were purchased from Charles River Laboratories and used at 8 to 10 weeks of age. Lipocalin-2^−/−^ (Lcn2^−/−^) mice were kindly provided by S. Akira, Osaka University, Japan, and crossed back to a pure C57BL/6N background for at least 20 generations. The mice were infected intraperitoneally (i.p.) with 500 CFU of Wt *S.* Typhimurium or an isogenic *S.* Typhimurium *qseC*::Frt-Kkan mutant (*qseC*) diluted in 200 µl PBS (26). For infections with *S.* Typhimurium *entC*, *feo*, or *sitABCD* mutant, mice were i.p. infected with 5,000 CFU. Mice were injected i.p. with 5 mg DA/kg of body weight dissolved in 200 µl PBS or an equal volume of PBS every 12 h and sacrificed after 72 h of infection (52). The mice that received LED209 (Cayman Chemical, MI, USA) were treated orally with 20 mg/kg LED209 dissolved in 300 µl of a solution containing 19.1% dimethyl sulfoxide (DMSO; Roth), 23% PEG 400 (Sigma-Aldrich), and 55.9% sodium bicarbonate (pH 9) and 2% Tween 80 (Sigma-Aldrich) 3 h before and 3 h after infection (53).

After termination of the experiment at 72 h, the bacterial load of organs was determined by plating serial dilutions of organ homogenates or whole blood on LB agar (Sigma-Aldrich, Vienna, Austria), and the number of bacteria calculated per mg of tissue was calculated. For survival studies, mice were infected i.p. with 500 CFU of *S.* Typhimurium and injected i.p. with DA or PBS every 12 h.

### Laboratory determinations.

Neutrophil counts were quantified using a Vet-ABC animal blood counter (scil animal care company GmbH, Viernheim, Germany). Determination of serum Tf was performed with an ELISA kit (Abcam) according to the manufacturer’s instructions. Serum iron was measured using the QuantiChrom iron assay kit (BioAssay Systems, Hayward, CA) according to the manufacturer’s instructions. Tf saturation was calculated by using serum Tf and iron concentrations. Analysis of tumor necrosis factor alpha (TNF-α) levels in serum was performed with an ELISA kit according to the manufacturer’s protocol (R&D Systems, Minneapolis, USA). Serum hepcidin was measured with a commercially available kit in exact accordance with the manufacturer’s protocol (Intrinsic LifeSciences).

### RNA extraction and quantitative real-time PCR (qRT-PCR).

Preparation of total RNA and quantification of mRNA expression by quantitative reverse transcription PCR were performed as previously described (49). Hypoxanthine phosphoribosyltransferase (HPRT) was used as a reference gene. Bacterial primers and probes have been described previously (54, 55).

### Western blot analysis.

Protein extraction and Western blotting were carried out as described previously using a rabbit Fpn1 antibody (1:400; Eurogentec, Liege, Belgium) and a rabbit actin antibody (1:1,000; Sigma-Aldrich) as a loading control (20).

### Flow cytometry analysis.

Spleens and livers were aseptically removed and cut. Liver samples were incubated in Liberase-containing RPMI medium for 45 min at 37°C. Afterward, organs were strained through a 40-μm nylon mesh. The single cell suspension was further diluted in PBS supplemented with sterile 2% FBS and 0.5% bovine serum albumin (Carl Roth, Karlsruhe, Germany) and costained with antibodies for flow cytometric analysis. Cells were first gated using FSC/SSC characteristics, and doublets were sequentially excluded by comparing FSC width and area signals. Red pulp macrophages (RPMs) were identified as CD45^+^, Lin^−^ (Lin = CD3, CD19, CD49b) Gr1^−^, CD11b^low/dim^, F4/80^high^. The antibodies used are listed in Text S1 in the supplemental material. Data were acquired on a Gallios (Beckman Coulter) flow cytometer and analyzed with FlowJo software (FlowJo LLC).

10.1128/mBio.02624-18.1 TEXT S1 Supplemental methods. Download Text S1, PDF file, 0.06 MB. Copyright © 2019 Dichtl et al. 2019 Dichtl et al. This content is distributed under the terms of the Creative Commons Attribution 4.0 International license.

### Statistical analysis.

Statistical analysis was performed using an SPSS statistical package. Significance was determined by unpaired two-tailed Student’s *t* test, when only two groups were compared, or Mann-Whitney test. Analysis of variance (ANOVA) combined with Bonferroni’s correction was used for all other experiments. A log-rank test was used to compare survival curves. Unless otherwise specified, data are depicted as lower quartile, median, and upper quartile (boxes) with minimum and maximum ranges or as means ± SEMs (bars). Generally, *P* values less than 0.05 were considered significant by any test.
