# Supplementary material for: Dopamine Is a Siderophore-Like Iron Chelator That Promotes Salmonella enterica Serovar Typhimurium Virulence in Mice
Source: mBio. 2019 Feb 5;10(1):e02624-18. doi: 10.1128/mBio.02624-18 (PMC6428752; doi:10.1128/mBio.02624-18)
Supplement: FIG S1 [file mBio.02624-18-sf001.pdf]

Supplementary Figure 1:

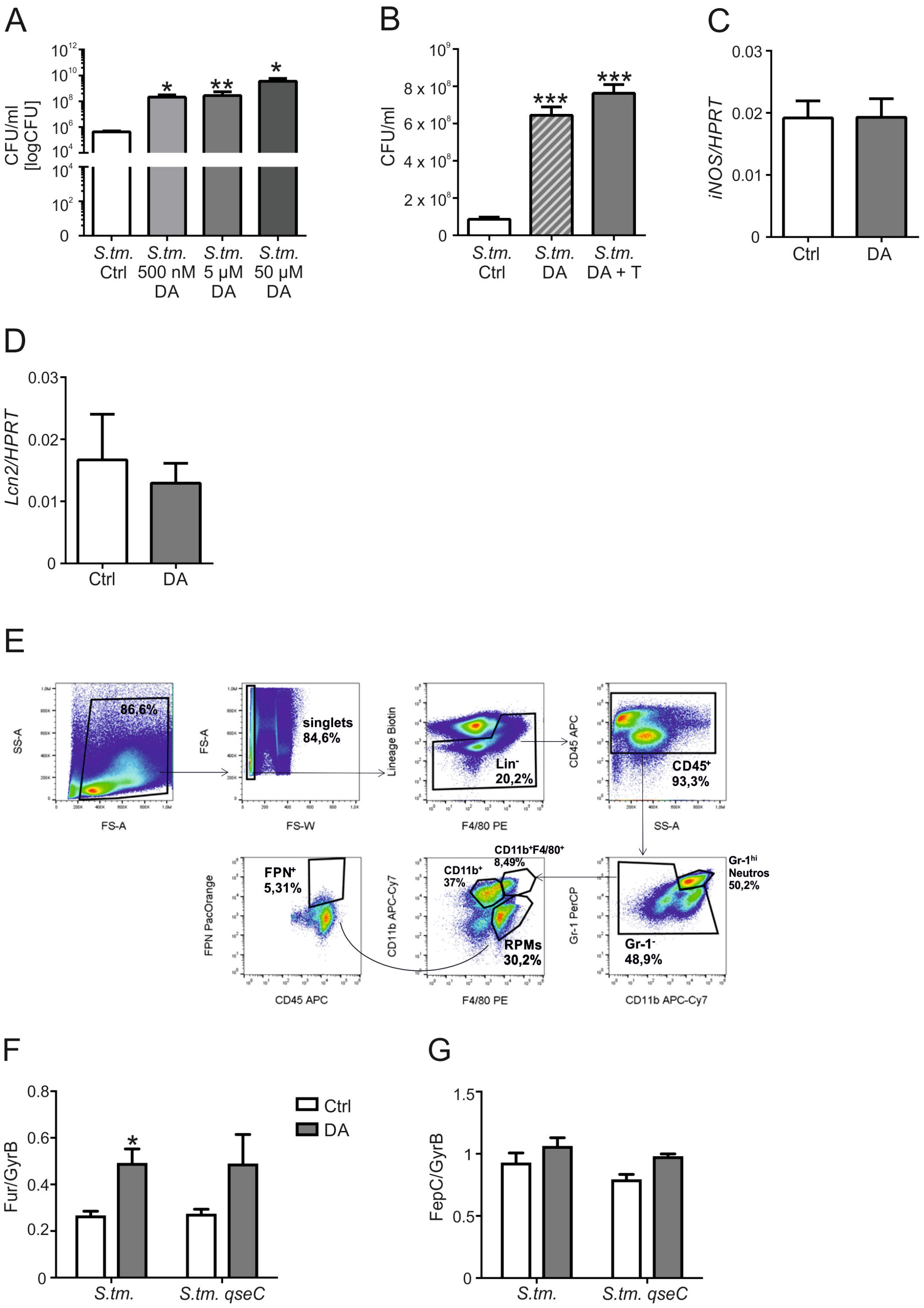

S. Typhimurium (*S*. tm.) was grown in the presence of 500 nM, 5 µM, 50 µM DA or solvent (Ctrl) for 12 h in DMEM medium and bacterial load determined by plating (n=2 independent experiments) (A). Values were log-transformed and the results compared by *t*-test. Superscripts indicate statistical significance compared to the control group. *S*. tm. was grown in the presence of 5 µM DA (*S*. tm. DA), 5 µM DA + 100 nM tranylcypromine (*S*. tm. DA + T) or solvent (Ctrl) for 12 h in DMEM medium and bacterial load determined by plating (n=2 independent experiments) (B). Healthy wt mice were injected with DA or solvent (Ctrl) every 12 h and spleens analyzed for *iNOS* (C) and *Lcn2* (D) expression. Data were normalized for mRNA levels of *HPRT*. Wt mice were injected with DA or solvent (Ctrl) every 12 h and, where indicated, i.p. infected with *S*. Typhimurium (*S*. tm.). Cells were first gated using FSC/SSC characteristics and doublets were sequentially excluded by comparing FSC-width and -area signals (E). Red pulp macrophages (RPMs) were identified as $CD45^+$, Lin^- (Lin = CD3, CD19, CD49b) $Gr1^-$, $CD11b^{low/dim}$, $F4/80^{high}$. *S*. Typhimurium (*S*. tm.) or an isogenic *qseC* mutant strain (*S*. tm. *qseC*) were grown in the presence of 5 µM DA or solvent (Ctrl) for 12 h in DMEM medium and the expression of bacterial iron metabolic genes measured by qRT-PCR. Expression of *fur* (F) and *fepC* (G) was determined relative to the housekeeping gene *gyrB*. Superscripts indicate statistical significance compared to the control group.
