# Supplementary material for: Dopamine Is a Siderophore-Like Iron Chelator That Promotes Salmonella enterica Serovar Typhimurium Virulence in Mice
Source: mBio. 2019 Feb 5;10(1):e02624-18. doi: 10.1128/mBio.02624-18 (PMC6428752; doi:10.1128/mBio.02624-18)
Supplement: TEXT S1 [file mBio.02624-18-s0001.pdf]

Supplementary Material and Methods

Flow Cytometry Analysis

For FACS analysis of murine macrophages, single-cell suspension of freshly isolated spleen and liver were collected. Cells were immunostained with the following antibodies:

PE-Fluor 610-anti-F4/80 (clone BM8, eBioscience), APC-anti-CD45 (clone A20, eBioscience), APC-Cyanine7-anti-CD11b (clone M1/70, eBioscience), PerCP-anti-Gr-1 (clone RB6-8C5, eBioscience), FITC-anti-Ly-6C (clone AL-21, BD Biosciences), PE-anti-CD71 (clone C2, BD Biosciences), Biotin-anti-CD49b (clone HMa2, eBioscience), Biotin-anti-CD19 (clone eBio1D3, eBioscience), Biotin-anti-CD3 (clone 17A2, BioLegend);

Fpn1 antibody was labeled with APEX Pacific Blue antibody labeling kit (Invitrogen). Secondary staining was accomplished with Streptavidin Pacific Orange conjugate (Invitrogen).

Cells were first gated using FSC/SSC characteristics, and doublets were excluded by comparing FSC-width and –area signals. Macrophages were identified as $CD45^{+}$, Lin^{-} (Lin = CD3, CD19, CD49b), $Gr1^{-}$, $CD11b^{low/dim}$, $F4/80^{high}$, Fpn^{+}.
